# Absence of germline mono-allelic promoter hypermethylation of the *CDH1 *gene in gastric cancer patients

**DOI:** 10.1186/1476-4598-8-63

**Published:** 2009-08-12

**Authors:** Hidetaka Yamada, Kazuya Shinmura, Masanori Goto, Moriya Iwaizumi, Hiroyuki Konno, Hideki Kataoka, Masami Yamada, Takachika Ozawa, Toshihiro Tsuneyoshi, Fumihiko Tanioka, Haruhiko Sugimura

**Affiliations:** 1 First Department of Pathology, Hamamatsu University School of Medicine, Hamamatsu, Japan; 2 Second Department of Surgery, Hamamatsu University School of Medicine, Hamamatsu, Japan; 3 Department of Gastroenterology, Hamamatsu Medical Center, Hamamatsu, Japan; 4 Department of Pathology, Hamamatsu Medical Center, Hamamatsu, Japan; 5 Department of Materials and Life Science, Shizuoka Institute of Science and Technology, Fukuroi, Japan; 6 Department of Pathology and Laboratory Medicine, Iwata City Hospital, Iwata, Japan

## Abstract

**Background:**

Germline mono-allelic promoter hypermethylation of the *MLH1 *or *MSH2 *gene in families with hereditary nonpolyposis colorectal cancer has recently been reported. The purpose of this study was to evaluate if germline promoter hypermethylation of the tumor suppressor gene *CDH1 *(*E-cadherin*) might cause predisposition to gastric cancer.

**Methods:**

We prepared two groups of samples, a group of blood samples from 22 patients with familial gastric cancer or early-onset gastric cancer selected from among 39 patients, and a group of non-cancerous gastric tissue samples from 18 patients with sporadic gastric cancer showing loss of CDH1 expression selected from among 159 patients. We then investigated the allele-specific methylation status of the *CDH1 *promoter by bisulfite sequencing of multiple clones.

**Results:**

Although there was a difference between the methylation level of the two alleles in some samples, there was no mono-allelic promoter hypermethylation in any of the samples.

**Conclusion:**

These results suggest that germline mono-allelic hypermethylation of the *CDH1 *promoter is not a major predisposing factor for gastric cancer.

## Background

Gastric cancer is one of the most common cancers worldwide, including in Japan, and gastric carcinogenesis is a multistep process in which environmental and genetic factors interact [1-6]. Among the genetic factors, the *CDH1 *gene, alternatively referred to as the *E-cadherin *gene, is one of the most important tumor suppressor genes in gastric cancer [6], and mutations, chromosomal deletions, and epigenetic modifications have been reported as mechanisms that cause CDH1 inactivation [6-17]. Somatic *CDH1 *mutations have been found in about 50% of diffuse-type gastric cancers [7], and germline *CDH1 *mutations have been reported in familial gastric cancers in several ethnic groups [6,8-13]. Promoter region hypermethylation, histone deacetylation, and chromatin condensation have been reported as epigenetic events in the *CDH1 *gene [14-17]. Among these mechanisms that cause CDH1 inactivation, inactivating germline mutations are the only genetic mechanism which is inherited in gastric cancer. Furthermore, since the germline *CDH1 *mutations have been found in only a certain percentage of familial gastric cancers, it is reasonable to hypothesize that CDH1 inactivation due to a previously unknown mechanism or inactivation of another gene plays a role in predisposing to gastric cancer. Interestingly, a germline mono-allelic hypermethylation of the *MLH1 *or *MSH2 *promoter has recently been reported in a subset of families with hereditary nonpolyposis colorectal cancer (HNPCC) [18-21]. Although mutations or chromosomal deletions in *MLH1 *and *MSH2 *have long been known to be hereditary genetic factors in HNPCC patients [22], such a germline epigenetic modification is a novel mechanism for the disease. Based on all of the above, we hypothesized that germline mono-allelic hypermethylation of the *CDH1 *promoter is responsible for gastric cancer, and in the present study we tested this hypothesis by examining the lesions of 39 patients with familial gastric cancer or early-onset gastric cancer. Furthermore, since germline *CDH1 *mutations are rarely found in gastric cancer patients with no family history of gastric cancer [23,24], we considered it worth examining patients with sporadic gastric cancer, even though the possibility of detecting germline mono-allelic hypermethylation of the *CDH1 *promoter may be small in that group.

## Methods

### Tissue samples, cell lines, and nucleic acid extraction

Although the diagnostic criteria for hereditary diffuse gastric cancer have been defined by the International Gastric Cancer Linkage Consortium [9], the Consortium also noted that the criteria should not be applied in Japan and Korea, where the background incidence of gastric cancer is high. In the present study we tentatively used the criterion "a proband and one or more cases of gastric cancer in the first-degree relatives" and called the cases collected familial gastric cancer. In this study "early-onset gastric cancer" is defined as gastric cancer diagnosed before 50 years of age. Blood samples were collected from 39 patients with familial gastric cancer or early-onset gastric cancer [12,25], and all of whom had been shown to be negative for germline *CDH1 *mutations [12,25]. Non-cancerous gastric tissue was collected from 159 gastric cancer patients treated at the Hamamatsu University Hospital. HeLa, HL-60, H358, and HT-29 cell lines were purchased from the American Type Culture Collection (Manassas, VA), and Lu-135 and MKN74 cell lines were purchased from Human Science Research Resource Bank (Osaka, Japan). Genomic DNA was extracted with a QIAamp DNA Blood Maxi Kit (QIAGEN, Valencia, CA) or with a DNeasy Tissue Kit (QIAGEN). Total RNA was extracted with an RNeasy Mini Kit (QIAGEN). The research protocol was approved by the Institutional Review Board of Hamamatsu University School of Medicine.

### Reverse transcription (RT)-polymerase chain reaction (PCR) analysis

Total RNA was converted to cDNA by using the SuperScript First-Strand Synthesis System for RT-PCR (Invitrogen, Carlsbad, CA) according to the manufacturer's instructions. PCR amplification was performed using the following sets of primers: 5'-AGA ACG CAT TGC CAC ATA CAC-3' and 5'-GAG GAT GGT GTA AGC GAT GG-3' for the CDH1 transcripts and 5'-CCA AGG TCA TCC ATG ACA AC-3' and 5'-CAC CCT GTT GCT GTA GCC A-3' for the GAPDH transcripts. PCR products were fractionated by electrophoresis on a 2.0% agarose gel and stained with ethidium bromide, and the gel was examined under UV light. A 100-bp DNA ladder (New England Biolabs, Beverly, MA) was used.

### Genotyping analysis

The -348_-347insA, -161C>A, and -73A>C polymorphisms in the *CDH1 *promoter region were genotyped by PCR-restriction fragment length polymorphism (RFLP) analysis. These polymorphisms are denoted based on the GenBank accession number NT_010498 (*CDH1 *reference sequence used in this study). The -161C>A polymorphism has also been reported as the -160C>A polymorphism in several previous papers [26]. The *CDH1 *promoter region was amplified by PCR with HotStarTaq DNA polymerase (QIAGEN) and the following set of primers: 5'-GCT ACT AGA GAG GCT GGG GC-3' and 5'-TCA CAG GTG CTT TGC AGT TC-3'. The PCR products were digested with *Bsm*AI for the -348_-347insA polymorphism and with *Bst*EII for the -161C>A and -73A>C polymorphisms. The digestion products were separated by electrophoresis on a 2.0% agarose gel and stained with ethidium bromide, and the gel was examined under UV light. To validate the results of the PCR-RFLP analysis, some PCR products exhibiting a different genotype in the PCR-RFLP analysis were randomly selected and directly sequenced with a BigDye Terminator Cycle Sequencing Reaction Kit (Applied Biosystems, Tokyo, Japan) and an ABI 3100 Genetic Analyzer (Applied Biosystems). Deviation of genotype distribution from the Hardy-Weinberg equilibrium (HWE) was tested with SNPAlyze software (Dynacom, Yokohama, Japan).

### Immunohistochemical analysis

Paraffin-embedded tissue sections were deparaffinized, rehydrated, and antigen-retrieved. The sections were then treated with 3% hydrogen peroxide to block endogenous peroxidase activity. Next, the sections were incubated with an anti-CDH1 monoclonal antibody (clone 36B5; Novocastra, Newcastle, UK), and then with dextran polymer conjugated with goat anti-mouse IgG and horseradish peroxidase (ChemMate Envision Kit, DAKO, Kyoto, Japan). The antigen-antibody complex was visualized with 3,3'-diaminobenzidine tetrahydrochloride and counterstained with hematoxylin. This analysis was performed with a DAKO autostainer (DAKO) [27]. Hematoxylin-eosin (H-E) stained slides were also prepared.

### Allele-specific methylation analysis

A 500 ng sample of genomic DNA was treated with sodium bisulfite using the EpiTect Bisulfite Kit (QIAGEN) according to the manufacturer's instructions. The *CDH1 *promoter region was amplified by PCR with HotStarTaq DNA polymerase (QIAGEN) and the bisulfite-treated DNA. The primers used were 5'-TTT TTT TTG ATT TTA GGT TTT AGT GAG-3' and 5'-ACT CCA AAA ACC CAT AAC TAA CC-3' for DNA extracted from the cell lines and 5'-TGG TGG TGT GTA TTT GTA TTT TTA GGA G-3' and 5'-ACT CCA AAA ACC CAT AAC TAA CC-3' for DNA extracted from blood or gastric tissue. The PCR conditions consisted of initial denaturation at 95°C for 15 min, 45 cycles of denaturation at 94°C for 30 sec, annealing at 59°C for 30 sec, and extension at 72°C for 1 min, and then a final extension at 72°C for 10 min. The PCR product was subcloned into a pGEM-T Easy vector (Promega, Madison, WI). At least 8 clones were sequenced with a BigDye Terminator Cycle Sequencing Reaction Kit (Applied Biosystems) and an ABI 3100 Genetic Analyzer (Applied Biosystems). The sequencing results and genotyping results for the -348_-347insA, -161C>A, or -73A>C polymorphisms described in "Genotyping analysis" section were used to evaluate allele-specific methylation status in the analysis of blood and gastric tissue.

## Results

### Inverse association between *CDH1 *promoter methylation level and CDH1 mRNA expression level in human cell lines

**Figure 1:**
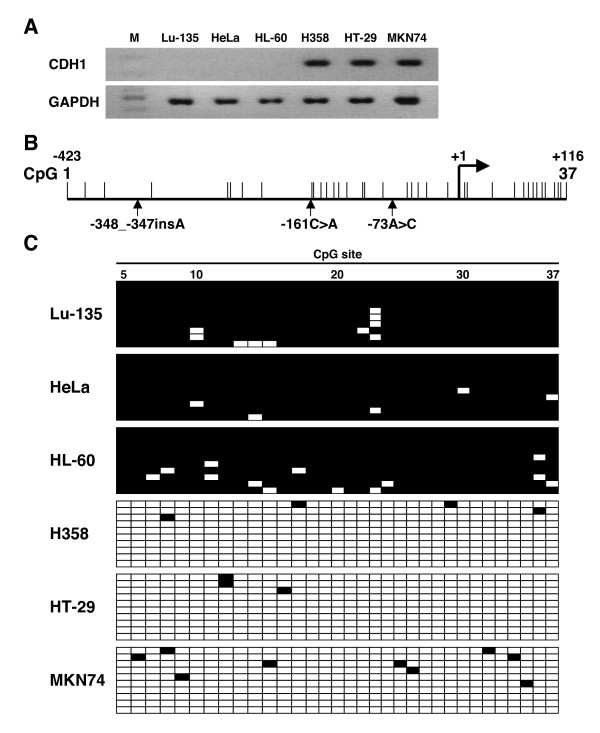
**Inverse association between *CDH1 *promoter methylation level and CDH1 mRNA expression level in human cell lines**. (A) Detection of the CDH1 mRNA transcripts in 6 human cell lines by reverse transcription (RT)-polymerase chain reaction (PCR) analysis. mRNA transcripts of the *GAPDH*, a housekeeping gene, were also amplified as an internal control. M, 100-bp DNA ladder. (B) Map of the CpG sites in the *CDH1 *promoter. The positions of the CpG sites are indicated by vertical lines. Vertical arrows indicate the location of the -348_-347insA, -161C>A, and -73A>C genetic polymorphisms. +1, transcription start site. (C) Determination of the methylation status of the CpG sites in the *CDH1 *promoter in 6 human cell lines by bisulfite sequencing analysis. Ten subcloned promoter fragments were sequenced in each cell line. Each horizontal row represents a single allele. The positions of the CpG sites are numbered at the top of the column. Methylated CpG sites are shown as black boxes and unmethylated CpG sites as white boxes.

First, we tried setting up an experimental system that could be used to evaluate the methylation status of the *CDH1 *promoter in human cells. Six human cell lines, Lu-135, HeLa, HL-60, H358, HT-29, and MKN74, were examined for mRNA expression by RT-PCR analysis, and the results showed no CDH1 expression in the Lu-135, HeLa, and HL-60 cells but strong CDH1 expression in the H358, HT-29, and MKN74 cells (Figure 1A). The status of CDH1 expression in the HeLa, HL-60, and HT-29 cells was compatible with previous reports [28,29], and the results in the other cell lines were novel findings. Next, the methylation status of 33 CpG sites in the *CDH1 *promoter region was examined in the six cell lines by bisulfite sequencing (Figure 1B). The results showed that the CpG sites in the *CDH1 *promoter were thoroughly methylated in the Lu-135, HeLa, and HL-60 cell lines, which did not express CDH1, but that they were almost completely unmethylated in the H358, HT-29, and MKN74 cell lines, which expressed CDH1 (Figure 1C). These results indicated that the *CDH1 *promoter methylation status of human cell lines as determined by our bisulfite sequencing analysis is inversely associated with the mRNA expression status of the *CDH1 *gene. They also indicated that the experimental system we set up to evaluate the methylation status of the *CDH1 *promoter was valid.

### Absence of germline mono-allelic hypermethylation in the *CDH1 *promoter in gastric cancer

**Figure 2:**
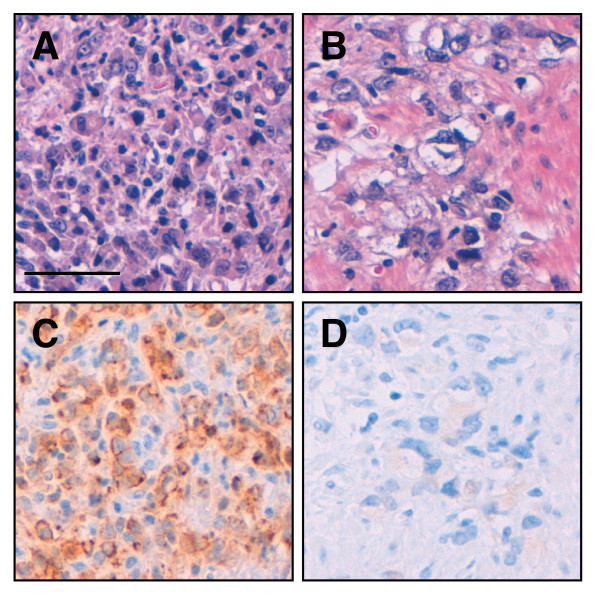
**Immunohistochemical examination of CDH1 protein expression in sporadic gastric cancers**. Representative gastric cancer samples are shown. (A) and (C), sporadic gastric cancer showing CDH1 protein expression; (B) and (D), sporadic gastric cancer not showing CDH1 expression. (A) and (B), H-E stained; (C) and (D), immunostained for CDH1. Scale bar, 50 μm.

**Figure 3:**
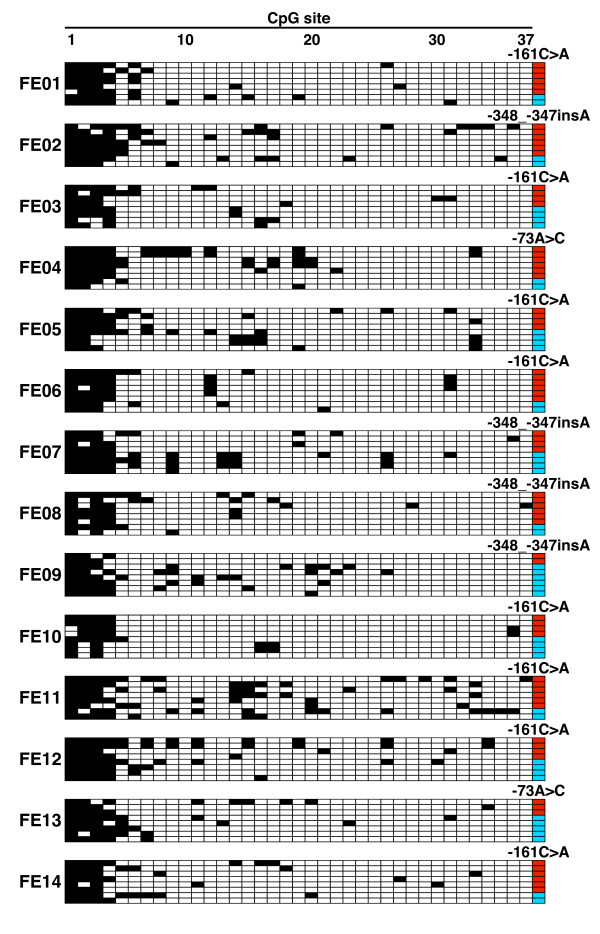
**DNA methylation patterns of the *CDH1 *promoter in the blood of patients with familial gastric cancer or early-onset gastric cancer determined by bisulfite sequencing analysis**. Eight subcloned promoter fragments were sequenced in each sample, and the results for 14 representative samples are shown. Each horizontal row represents a single allele. The positions of the CpG sites are numbered at the top of the column. Methylated CpG sites are shown as black boxes, and unmethylated CpG sites as white boxes. The far right column indicates the allele of the -348_-347insA, -161C>A, or -73A>C polymorphism: wild-type allele, red; variant-type allele, sky blue.

**Figure 4:**
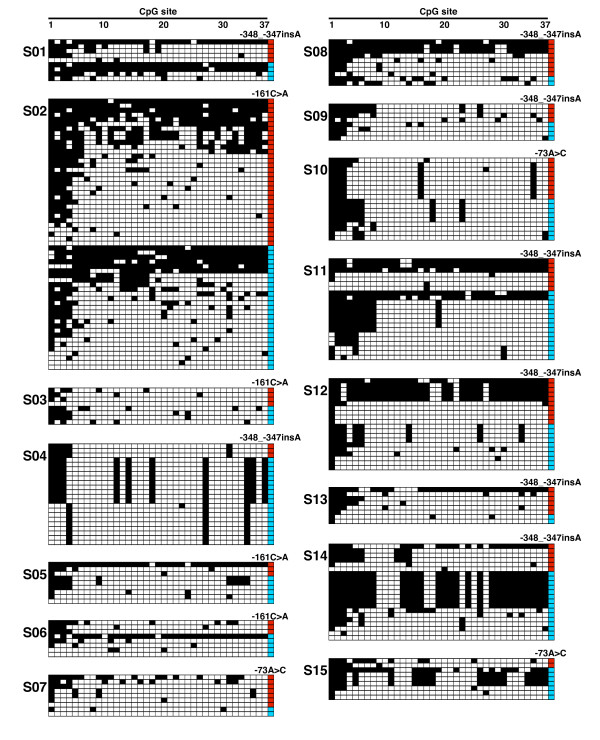
**DNA methylation patterns of the *CDH1 *promoter in non-cancerous gastric tissue from patients with sporadic gastric cancer determined by bisulfite sequencing analysis**. At least 8 subcloned promoter fragments were sequenced in each sample, and the results for 15 representative samples are shown. Each horizontal row represents a single allele. The positions of the CpG sites are numbered at the top of the column. Methylated CpG sites are shown as black boxes, and unmethylated CpG sites as white boxes. The far right column indicates the allele of the -348_-347insA, -161C>A, or -73A>C polymorphism: wild-type allele, red; variant-type allele, sky blue.

We tried investigating the allele-specific methylation status of the *CDH1 *promoter in two groups of gastric cancers, a familial gastric cancer or early-onset gastric cancer group and a sporadic gastric cancer group. Since information on the genetic polymorphisms within the *CDH1 *promoter region is useful for discriminating the *CDH1 *alleles subcloned in the bisulfite sequencing analysis, the -348_-347insA, -161C>A, and -73A>C polymorphisms in the *CDH1 *promoter region were genotyped by PCR-RFLP analysis (Table 1). All the genotyping results were in HWE (*P *> 0.05). The DNA in the blood of 39 patients with familial gastric cancer or early-onset gastric cancer was genotyped and the 22 patients with at least one heterozygous promoter polymorphism were selected. Loss of CDH1 protein expression identified by CDH1 immunohistochemical analysis, in addition to selection by polymorphism genotyping, was used to select the sporadic group (Figure 2). As a result, 18 patients with sporadic gastric cancer showing loss of CDH1 protein expression were ultimately chosen from a total of 159 patients. We then tested DNA from the blood of the 22 patients with familial gastric cancer or early-onset gastric cancer and DNA from non-cancerous gastric tissue of the 18 patients with sporadic gastric cancer to determine the methylation status of the *CDH1 *promoter region by bisulfite sequencing analysis. Various percentages of methylation of CpG sites in the *CDH1 *promoter were detected, but the nearly complete methylation of the sites observed in the analysis of the cell lines was not found in any of the samples (Figure 3 and 4). Interestingly, there were differences between the methylation level of the two *CDH1 *alleles in some non-cancerous gastric tissue samples (e.g., S12 and S14 in Figure 4), but the clear mono-allelic hypermethylation observed in the *MLH1 *promoter of the HNPCC patients [18-20] was not observed in the *CDH1 *promoter in any of the samples (Figure 3 and 4). These results suggest that germline mono-allelic hypermethylation of the *CDH1 *promoter is not a major predisposing factor for gastric cancer.

**Table 1:** Distribution of the genotypes of the three *CDH1 *promoter polymorphisms in gastric cancer patients

Genetic polymorphism^a^	dbSNP ID^b^	Samples^c^	Number of patients with a heterozygous genotype (%)	Number of patients homozygous for a varinat allele (%)	Variant allele frequency
-348_-347insA	rs5030625	F/EGC	9 (23.1%)	4 (10.3%)	21.8%
		SGC	55 (34.6%)	15 (9.4%)	26.7%
-161C>A	rs16260	F/EGC	11 (28.2%)	0 (0.0%)	14.1%
		SGC	51 (32.1%)	7 (4.4%)	20.4%
-73A>C	rs28372783	F/EGC	5 (12.8%)	0 (0.0%)	6.4%
		SGC	19 (11.9%)	0 (0.0%)	6.0%

a Nucleotide +1 is the transcription start site. The reference sequence is accession number NT_010498.

b Identification number registered in the database of single nucleotide polymorphisms (dbSNP) homepage of the National Center for Biotechnology Information web site .

c F/EGC, familial gastric cancer or early-onset gastric cancer; SGC, sporadic gastric cancer.

## Discussion

In this study blood from 22 patients with familial gastric cancer or early-onset gastric cancer and non-cancerous gastric tissues from 18 patients with sporadic gastric cancer showing loss of CDH1 expression was analyzed for allele-specific methylation status of the *CDH1 *promoter by bisulfite sequencing analysis. Although a difference between the methylation level of the two *CDH1 *alleles was found in some non-cancerous gastric tissue samples, no mono-allelic promoter hypermethylation of the *CDH1 *gene was detected in any of them. This finding suggests that germline promoter hypermethylation of the *CDH1 *gene is not involved in any mechanism that causes susceptibility to gastric cancer. This is the first report of an investigation into whether germline mono-allelic hypermethylation of the *CDH1 *promoter is a predisposing factor for gastric cancer.

Since bisulfite allelic sequencing enables detailed, base-by-base measurement of CpG methylation and discrimination between the wild-type and variant type at the polymorphism site, we used it to determine methylation status in the *CDH1 *promoter region. The results showed that *CDH1 *promoter methylation status determined by the bisulfite sequencing analysis was inversely associated with mRNA expression status of the *CDH1 *gene in the human cell lines, indicating that a valid system had been established. However, no mono-allelic hypermethylation of the *CDH1 *promoter was found in any of the samples, suggesting that it is not a predisposing factor for gastric cancer. Caution is required, however, since the absence of germline mono-allelic promoter hypermethylation in this study may have been due to the limited number of patients analyzed and Hitchins *et al*. reported a very low incidence of germline mono-allelic hypermethylation of the *MLH1 *promoter in HNPCC (1 out of 160 HNPCC candidates) [19], it is impossible to rule out the possibility that analysis of a larger number of gastric cancer patients would reveal cases with germline mono-allelic hypermethylation of the *CDH1 *promoter. However, there were two reasons for the limited number of patients utilized for the allele-specific methylation analysis in our study. One reason is that the incidence of familial gastric cancer or early-onset gastric cancer is relatively low, and the other is that selection in regard to *CDH1 *promoter polymorphisms and CDH1 expression was necessary in this study. We therefore think that the results of our investigation of these selected samples are very important. Since all of the patients with familial gastric cancer or early-onset gastric cancer whose samples we analyzed had already been shown to be negative for germline *CDH1 *mutation [12,25], genetic or epigenetic events in other genes may have been involved in the gastric carcinogenesis in those patients.

Although no mono-allelic hypermethylation of the *CDH1 *promoter was detected in this study, various levels of methylation of CpG sites in the *CDH1 *promoter were detected in all of the samples. This finding is compatible with findings reported previously [30,31] and may be related to aging or *Helicobacter pylori *infection as reported in those papers [30,31]. Interestingly, there were differences between the two alleles in methylation level of the *CDH1 *promoter in some non-cancerous gastric tissue samples. Allele-specific methylation of the *CDH1 *promoter has been reported to be the second genetic hit in gastric cancer tissue from patients with familial gastric cancer [15], but the mechanism underlying the *CDH1 *allele-specific methylation has not been elucidated in either non-cancerous or cancerous gastric tissue. Future investigation of this point should improve our understanding of gastric carcinogenesis.

Since germline mono-allelic promoter hypermethylation and transgenerational inheritance of such an epigenetic event is a recent finding in humans, there have been only a small number of papers documenting the germline epigenetic modification. Heritable germline mono-allelic hypermethylation of the *MLH1 *or *MSH2 *gene has been found in a subset of families with HNPCC [18-21]. On the other hand, similar investigation of the *APC *gene in patients with familial adenomatous polyposis (FAP), attenuated FAP, and hyperplastic polyposis, of the *BRCA1 *gene in patients with familial breast cancer, and of the *CDKN2A *gene in patients with familial melanoma have revealed no germline mono-allelic promoter hypermethylation of any of these genes [32-34]. Based on these findings and the results of our own study, germline mono-allelic hypermethylation is unlikely to be present in all genes responsible for hereditary cancer syndromes. Thus, it will be important to identify genes with germline epigenetic modifications in the future, because the results will be useful in making a precise diagnosis and in conducting surveillance and management of cancer patients and their family members.

In this study the distribution of the genotypes of the three *CDH1 *promoter polymorphisms was in HWE, and the results of PCR-RFLP analysis were confirmed by direct sequencing in some samples. This means our genotyping was performed properly, and proper genotyping was important to selecting the gastric cancer patients in our study. Since there is a *CDH1 *haplotype associated with increased gastric cancer risk and the haplotype contains the -161A allele [3], the genotyping method will be also valuable for evaluating the risk of gastric cancer.

There are two main histopathological types of gastric cancer, a diffuse-type and an intestinal-type. The 5-year survival rate has been reported to be lower for diffuse-type gastric cancer than for intestinal-type gastric cancer, and the incidence of peritoneal recurrence has been reported to be higher in diffuse-type gastric cancer than in intestinal-type gastric cancer [35,36]. Although no germline mono-allelic hypermethylation of the *CDH1 *promoter was found in this study, we believe it is important to continue to evaluate *CDH1 *gene status from various standpoints, because CDH1 inactivation is closely related to the pathogenesis of diffuse-type gastric cancer.

## Conclusion

The present results suggest that germline mono-allelic hypermethylation of the *CDH1 *promoter is not a major predisposing factor for gastric cancer.

## Competing interests

The authors declare that they have no competing interests.

## Authors' contributions

HY carried out the experiments and drafted the manuscript. KS, HK, and HS conceived of the study, participated in its design and coordination and helped to draft the manuscript. MG and TT participated in a part of the experiments. MI, HK, HK, MY, TO, and FT provided the samples needed for this study. All authors read and approved the final manuscript.
